# Fecal Extracellular Vesicle Metabolomics as a Non-Invasive Biomarker Source in Colorectal Cancer: TPOT AutoML Superiority over Tree-Based Models with SHAP and LIME Clinical Interpretability

**DOI:** 10.3390/ijms27125451

**Published:** 2026-06-16

**Authors:** Fatma Hilal Yagin, Yavuz Korkmaz, Cemil Colak, Fahaid Al-Hashem, Sarah A. Alzakari, Amal K. Alkhalifa, Mohammadreza Aghaei

**Affiliations:** 1 Department of Biostatistics, Faculty of Medicine, Malatya Turgut Ozal University, Malatya 44210, Türkiye; 2 Department of Family Medicine, Faculty of Medicine, Malatya Turgut Ozal University, Malatya 44210, Türkiye; 3 Department of Biostatistics, and Medical Informatics, Faculty of Medicine, Inonu University, Malatya 44280, Türkiye; cemil.colak@inonu.edu.tr; 4 Department of Physiology, College of Medicine, King Khalid University, Abha 61421, Saudi Arabia; 5 Department of Computer Sciences, College of Computer and Information Sciences, Princess Nourah bint Abdulrahman University, P.O. Box 84428, Riyadh 11671, Saudi Arabia; 6 Department of Ocean Operations and Civil Engineering, Norwegian University of Science and Technology (NTNU), 6009 Ålesund, Norway; 7 Department of Sustainable Systems Engineering (INATECH), Albert Ludwigs University of Freiburg, 79110 Freiburg, Germany

**Keywords:** colorectal cancer, fecal metabolomics, extracellular vesicles, explainable artificial intelligence, SHAP, LIME, biomarker discovery, TPOT

## Abstract

Colorectal cancer (CRC) remains one of the leading causes of cancer-related mortality worldwide, highlighting the critical need for non-invasive, accurate, and interpretable diagnostic tools. Metabolomic profiling of fecal microbial extracellular vesicles (EVs) offers a promising yet underexplored avenue for biomarker discovery when integrated with explainable machine learning (ML) frameworks. This study aimed to identify stool-derived microbial EV metabolite biomarkers that discriminate CRC patients from healthy controls and to develop interpretable ML classifiers for non-invasive CRC detection. Metabolomic profiles of fecal microbial EVs from 76 age- and sex-comparable participants (36 CRC, 40 controls) were obtained using LC/QTOFMS and GC/TOFMS. Three ML classifiers (TPOT, LightGBM, XGBoost) were trained and evaluated through 100-repeat stratified hold-out and nested 5-fold cross-validation, with SHAP and LIME applied for global and local interpretability. Fourteen metabolites were significantly dysregulated between the CRC and control groups (adjusted *p* < 0.05), with 13 upregulated and one (aminoisobutyric acid) downregulated. Furoic acid exhibited perfect diagnostic discrimination, followed by palmitic acid and tyramine. Nested cross-validation demonstrated robust performance: TPOT achieved AUC = 0.997 ± 0.005, sensitivity = 0.973 ± 0.022, and MCC = 0.957 ± 0.033. Hold-out validation corroborated these findings (AUC = 0.998 ± 0.008). SHAP analysis identified furoic acid, palmitic acid, and tyramine as the dominant predictive features, while aminoisobutyric acid exhibited a distinctive protective pattern. LIME analysis corroborated these findings at the individual prediction level. The identified fecal EV-derived metabolite panel—particularly furoic acid, palmitic acid, and tyramine—shows strong potential to predict CRC in a non-invasive, interpretable manner; however, given the modest sample size, these findings should be considered hypothesis-generating and require validation in larger, prospective, multi-center cohorts before clinical translation.

## 1. Introduction

Colorectal cancer (CRC) constitutes one of the most prevalent and lethal malignancies globally, ranking as the third most commonly diagnosed cancer and the second leading cause of cancer-related death worldwide [1,2]. Despite considerable advances in therapeutic strategies, survival outcomes remain heavily dependent on the stage at which the disease is detected, with five-year survival rates exceeding 90% for localized disease but declining sharply to approximately 14% for distant metastases [3]. These figures highlight the critical importance of early detection in improving patient prognosis and reducing the overall burden of CRC.

Colonoscopy remains the gold standard for CRC screening; however, its invasive nature, requirement for bowel preparation, procedural discomfort, and substantial healthcare costs have contributed to suboptimal screening adherence, particularly in resource-limited settings. Similarly, widely adopted non-invasive stool-based screening tests—such as the fecal immunochemical test (FIT) and the guaiac-based fecal occult blood test (gFOBT)—suffer from limited sensitivity for early-stage lesions and precancerous adenomas [4]. These limitations have motivated an active search for novel, non-invasive biomarkers capable of achieving both high sensitivity and high specificity for CRC detection [5,6].

Metabolomics—the comprehensive analysis of low-molecular-weight metabolites and their intermediates within a biological system—has emerged as a particularly promising approach [7,8]. Tumor cells are characterized by profound metabolic reprogramming that extends beyond glucose metabolism to encompass lipid, amino acid, and nucleotide biosynthetic pathways [9,10]. These metabolic alterations generate distinctive biochemical signatures that can be detected in accessible biofluids—including blood, urine, and stool—thereby offering a window into the underlying pathophysiology of CRC without the need for invasive tissue sampling.

Among the various biological matrices available for biomarker discovery, stool specimens hold a distinct advantage for detecting CRC-specific metabolic signatures. The fecal metabolome is shaped not only by host metabolic processes but also by the gut microbiome, which has been increasingly recognized as a critical modulator of colorectal carcinogenesis [11]. Fecal microbial extracellular vesicles (EVs)—nano-sized membrane-bound particles secreted by gut bacteria—offer an especially compelling analytical target. These vesicles encapsulate a diverse array of metabolites, proteins, and nucleic acids that reflect the functional state of the microbial community and its interactions with the host epithelium [12]. Consequently, the metabolomic profiling of stool-derived microbial EVs has the potential to capture disease-relevant metabolic perturbations with greater specificity than bulk fecal analysis alone.

In parallel with advances in metabolomics, machine learning (ML) has substantially advanced biomarker discovery and disease classification by enabling the construction of predictive models capable of identifying complex, non-linear patterns within high-dimensional omics data [13]. Ensemble learning algorithms such as eXtreme Gradient Boosting (XGBoost; [14]) and Light Gradient Boosting Machine (LightGBM; [15]) have shown particular effectiveness in navigating the sparse, heterogeneous feature spaces characteristic of metabolomics datasets. Furthermore, automated machine learning (AutoML) platforms—exemplified by the Tree-based Pipeline Optimization Tool (TPOT)—leverage genetic programming to systematically explore and optimize the entire predictive pipeline, thereby reducing human bias and increasing reproducibility [16].

Despite the impressive predictive performance of these ML algorithms, their clinical translation is frequently hindered by a lack of model interpretability—commonly referred to as the ‘black-box’ problem. In the context of medical decision-making, clinicians and regulatory bodies increasingly demand that predictive models not only achieve high accuracy but also provide transparent, human-understandable explanations for their predictions [17,18]. Explainable artificial intelligence (XAI) techniques have been developed to address this imperative. SHapley Additive exPlanations (SHAP) provides a theoretically grounded, game-theory-based framework for quantifying the global and local contribution of each feature to model output [19], whereas Local Interpretable Model-Agnostic Explanations (LIME) generates local surrogate models that approximate the behavior of complex classifiers in the vicinity of individual predictions [20]. The complementary application of both techniques enables a comprehensive assessment of model behavior at multiple levels of granularity.

Despite the promise of metabolomics-based approaches, no prior study has directly compared automated ML pipelines (TPOT) against gradient-boosting algorithms for CRC classification using fecal microbial EV metabolomics, nor have they applied dual-layer XAI (SHAP + LIME) to this specific matrix. The specific hypotheses of this study were: (i) that a panel of stool-derived EV metabolites can discriminate CRC from healthy controls with AUC > 0.90, (ii) that TPOT would outperform manually tuned gradient-boosting models, and (iii) that SHAP and LIME would reveal biologically coherent directional effects for identified metabolites.

The main aim of this study is to determine whether the metabolomic cargo of fecal microbial extracellular vesicles can serve as a non-invasive, interpretable biomarker source for the discrimination of CRC patients from healthy controls. To achieve this aim, the study pursues four specific objectives: (i) performing comprehensive metabolomic profiling of stool-derived microbial EVs in a cohort of CRC patients and healthy controls; (ii) identifying differentially expressed metabolites and evaluating their individual diagnostic performance via receiver operating characteristic (ROC) analysis; (iii) developing and comparing three optimized ML classifiers—TPOT, LightGBM, and XGBoost—for CRC prediction; and (iv) applying SHAP and LIME to the best-performing model to elucidate the magnitude, direction, and biological plausibility of metabolite contributions to CRC classification.

## 2. Results

### 2.1. Differentially Expressed Metabolites and Individual Diagnostic Performance

**Figure 1:**
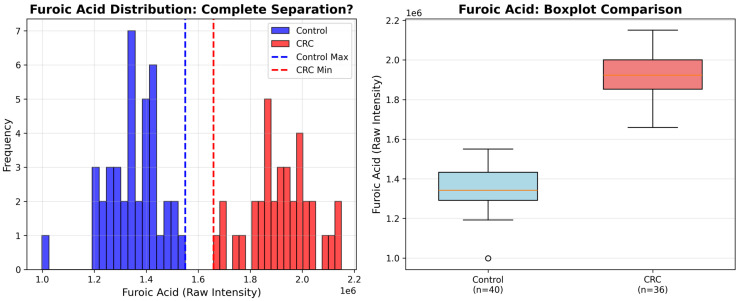
Distribution of furoic acid intensities demonstrating complete separation between CRC patients and healthy controls.

Fold change analysis identified 14 metabolites that were significantly dysregulated between the CRC and control groups following FDR correction (adjusted *p* < 0.05). Of these, 13 metabolites were upregulated and one—aminoisobutyric acid—was downregulated in the CRC group relative to healthy controls (Table 1). Among the most prominently elevated metabolites, succinic acid demonstrated the highest fold change (FC = 4.523), followed by oxalic acid (FC = 4.038). Both metabolites exhibited substantial upregulation and highly significant adjusted *p*-values (*p* < 0.001), suggesting marked perturbations in organic acid metabolism. Isoleucine and leucine, two branched-chain amino acids (BCAAs), were also significantly elevated (FC = 2.149 and 2.059, respectively; *p* < 0.001), indicating dysregulation of amino acid metabolic pathways. In terms of statistical significance, furoic acid and palmitic acid yielded the highest −log10(*p*) values (13.146 and 12.940, respectively), reflecting the most statistically consistent between-group differences in the cohort. Tyramine similarly demonstrated strong significance (−log10(*p*) = 12.200; FC = 1.592; *p* < 0.001). The sole downregulated metabolite, aminoisobutyric acid, exhibited a modest but statistically significant reduction (FC = 0.947; Log2FC = −0.079; adjusted *p* = 0.006). ROC curve analysis was performed for each of the 14 significant metabolites to assess their individual diagnostic utility (Table 1). Furoic acid emerged as the single most discriminative biomarker, achieving a perfect AUC of 1.000 (95% CI: 1.000–1.000) with both sensitivity and specificity of 1.000, reflecting complete separation between groups with no distributional overlap (Figure 1). Palmitic acid demonstrated comparably outstanding performance (AUC = 0.996; 95% CI: 0.986–1.000; sensitivity = 1.000; specificity = 0.925), followed by tyramine (AUC = 0.981; 95% CI: 0.948–1.000) and phenol (AUC = 0.966; 95% CI: 0.928–0.994). Oxalic acid and succinic acid—the two metabolites with the highest fold changes—also displayed strong discriminatory performance (AUC = 0.898 and 0.845, respectively). Conversely, lysine (AUC = 0.665) and ethanolamine (AUC = 0.694) showed more modest individual discriminatory power, suggesting they may contribute more meaningfully as part of a multi-metabolite panel rather than as standalone markers.

### 2.2. Machine Learning Classification Performance

All three ML algorithms were evaluated across 100 repeated stratified hold-out validations (Table 2). TPOT achieved the highest overall performance, with a mean AUC of 0.998 ± 0.008, accuracy of 0.981 ± 0.036, precision of 0.986 ± 0.040, sensitivity of 0.976 ± 0.055, F1-score of 0.980 ± 0.037, Brier score of 0.026 ± 0.038, and MCC of 0.963 ± 0.069, confirming its superiority as an automated pipeline optimization framework for this metabolomics classification task.

XGBoost demonstrated comparable performance, yielding a mean AUC of 0.997 ± 0.012, accuracy of 0.976 ± 0.040, sensitivity of 0.964 ± 0.069, and MCC of 0.955 ± 0.074, with consistently low Brier scores (0.034 ± 0.029) indicating reliable probabilistic calibration. The narrow standard deviations observed for both TPOT and XGBoost across all metrics reflect stable generalization across repeated random partitions of the dataset.

LightGBM exhibited greater variability across repetitions, with a mean AUC of 0.855 ± 0.228, accuracy of 0.846 ± 0.223, and MCC of 0.693 ± 0.447. The substantially higher standard deviations relative to TPOT and XGBoost suggest sensitivity to the specific training partition composition under the applied regularization constraints, a known limitation of gradient boosting methods in small-sample settings where hyperparameter optimization may converge to suboptimal configurations in certain random splits. Despite this variability, LightGBM achieved perfect or near-perfect classification in the majority of repetitions, as reflected by median performance substantially above the reported means.

Taken together, these results indicate that the identified fecal EV metabolite panel enables highly accurate CRC discrimination, with TPOT emerging as the most robust and best-performing pipeline under automated optimization, followed closely by XGBoost.

The 100-repeat nested CV AUC for TPOT was 0.997 (±0.005), compatible with the reported 0.998 from hold-out validation (Table 2). We report the nested CV estimates (Table 3) as our primary performance metrics to align with TRIPOD recommendations [21].

### 2.3. SHAP-Based Feature Importance and Directional Effects

**Figure 2:**
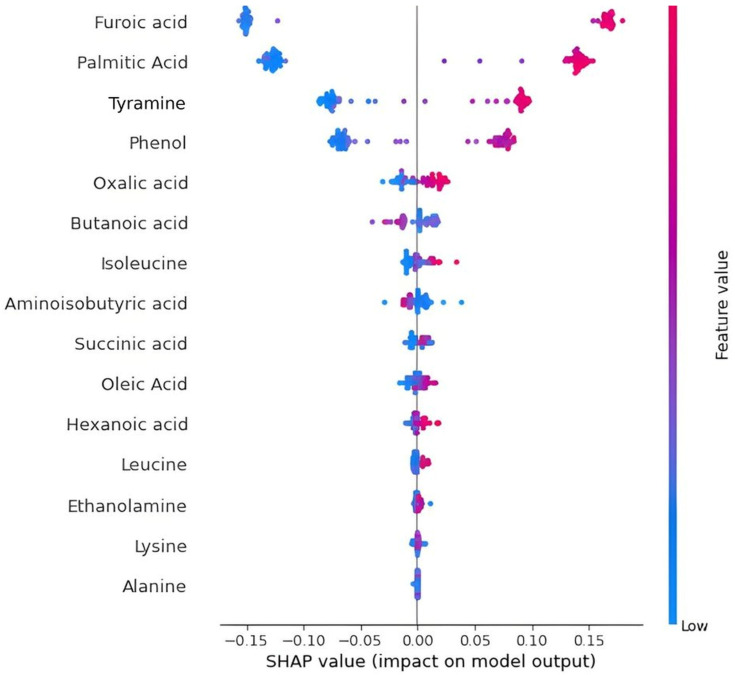
SHAP beeswarm plot illustrating the contribution of each metabolite to the classification model output selected by the TPOT pipeline. Each dot represents one sample; horizontal position reflects the magnitude and direction of the SHAP value; color gradient encodes observed feature value (pink = high concentration, blue = low concentration). Features are ranked in descending order of mean absolute SHAP value.

To enhance the interpretability of the best-performing model, SHAP analysis was performed on the underlying classifier selected by the TPOT pipeline, enabling quantification of the magnitude and directional contribution of each metabolite to individual predictions. The resulting beeswarm plot (Figure 2) illustrates the distribution of SHAP values for all input metabolites across the study cohort, including butanoic acid which was present in the dataset but did not reach the FDR-corrected significance threshold, where the horizontal position of each data point reflects the impact on model output and the color gradient indicates the observed feature value (pink = high concentration; blue = low concentration).

Furoic acid emerged as the most influential predictor, demonstrating the largest absolute SHAP values across samples. Elevated furoic acid concentrations were strongly associated with large positive SHAP values, indicating that high furoic acid significantly increases the predicted probability of CRC classification. Conversely, low furoic acid concentrations were associated with negative SHAP values, confirming that its absence exerts a protective effect. Palmitic acid ranked as the second most impactful feature, with high concentrations consistently producing strongly positive SHAP values, thereby serving as a robust positive predictor of CRC. A similar directional pattern was observed for tyramine, where elevated concentrations were associated with markedly positive SHAP values.

Phenol exhibited a pattern whereby high concentrations contributed positively to the model output—that is, elevated phenol levels were associated with increased likelihood of CRC classification. Oxalic acid and succinic acid displayed relatively concentrated SHAP distributions near zero, suggesting more moderate and consistent contributions, though their upregulation still directionally supported CRC prediction.

In contrast, aminoisobutyric acid demonstrated a distinctive inverse pattern: elevated concentrations were associated with negative SHAP values, indicating that high aminoisobutyric acid reduces the predicted probability of CRC. This observation is consistent with its downregulation in the patient group and suggests that aminoisobutyric acid may represent a protective metabolite whose depletion is mechanistically relevant to colorectal carcinogenesis. Isoleucine and leucine showed modest positive SHAP contributions, further corroborating the dysregulation of BCAA metabolism in this context. Metabolites including ethanolamine, lysine, alanine, hexanoic acid, oleic acid, and butanoic acid contributed minimally to the model output, with SHAP values clustered tightly around zero, implying limited independent discriminatory power despite reaching statistical significance in univariate analysis.

### 2.4. LIME-Based Local Interpretability Analysis

**Figure 3:**
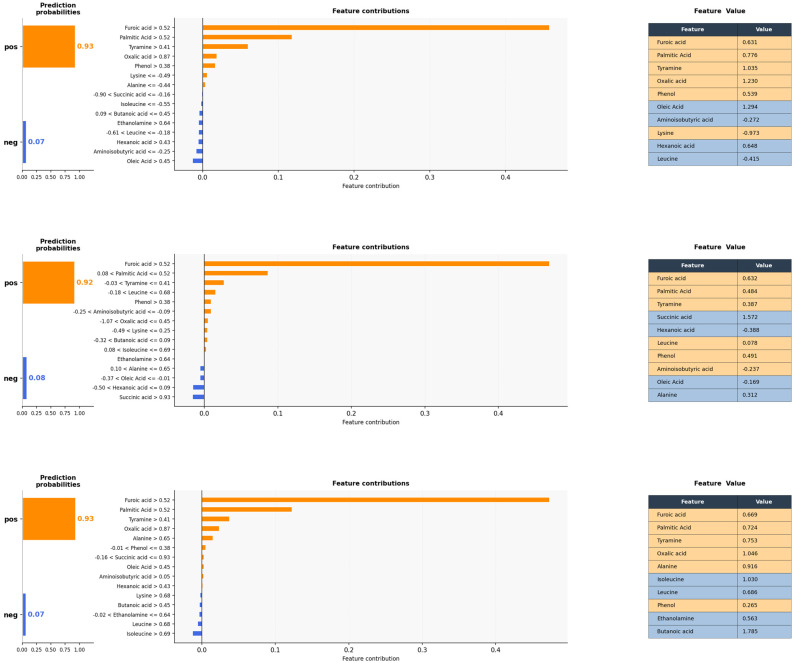
LIME Local Interpretability Analysis—Three Correctly Classified CRC Patients. Preprocessing: KNN imputation (k = 5) → log2 transformation → Pareto scaling applied within each fold (train set only); n_perturbations = 5000 per instance. Left panel: Predicted class probabilities (pos = CRC, neg = Control). Middle panel: Feature contributions—orange bars indicate positive contribution to CRC classification; blue bars indicate negative contribution (toward Control classification). Right panel: Feature values in scaled space—orange shading indicates high metabolite concentration; blue shading indicates low metabolite concentration.

To complement the global explanations provided by SHAP, LIME analysis was applied to three correctly classified CRC patients to characterize feature contributions at the individual prediction level (Figure 3). Across all three representative cases, the TPOT-selected classifier assigned CRC classification probabilities of 0.93, 0.92, and 0.93, respectively, with corresponding control probabilities of 0.07, 0.08, and 0.07, confirming high-confidence correct classifications. Furoic acid emerged as the dominant positive contributor in all three patients, consistent with its status as the most influential global predictor identified by SHAP analysis. Palmitic acid consistently ranked as the second most impactful feature across all three patients, with elevated concentrations driving CRC classification in each instance. Tyramine contributed positively in patients 1 and 3, while its contribution was absent in patient 2, suggesting some inter-individual variability in its predictive weight despite consistent upregulation at the cohort level. In terms of negative contributors—features directing the prediction toward the control class—aminoisobutyric acid and leucine exerted opposing effects in patient 1, consistent with their established downregulation and modest positive SHAP patterns, respectively. In patient 2, succinic acid and hexanoic acid contributed negatively, suggesting that within the local neighborhood of this particular instance, their concentration ranges overlapped with control-like patterns. In patient 3, isoleucine and leucine were identified as the primary negative contributors, despite both metabolites being globally upregulated in the CRC group, illustrating the capacity of LIME to capture locally non-monotonic feature behaviors that may be obscured in global importance analyses.

## 3. Discussion

The present study demonstrates that the integration of fecal microbial EV metabolomics with explainable ML classifiers achieves accurate and transparent non-invasive CRC detection. By combining comprehensive metabolomic profiling with three optimized ensemble learning algorithms and dual-layer XAI interpretability via SHAP and LIME, this work addresses a critical research area at the intersection of metabolomics-based biomarker discovery and transparent predictive modeling in oncology diagnostics.

The identification of 14 significantly dysregulated metabolites between CRC patients and healthy controls aligns well with what is currently known about CRC-associated metabolic alterations. Succinic acid, which exhibited the highest fold change in our cohort (FC = 4.523), is a key intermediate of the tricarboxylic acid (TCA) cycle. Accumulation of succinate has been linked to oncometabolite activity through its capacity to inhibit prolyl hydroxylase domain enzymes, thereby stabilizing hypoxia-inducible factor 1-alpha (HIF-1α) and promoting tumor angiogenesis, metabolic reprogramming, and immune evasion [22,23]. The substantial upregulation of oxalic acid (FC = 4.073) similarly aligns with prior reports of altered organic acid metabolism in CRC, although the precise mechanistic role of oxalate in colorectal carcinogenesis requires further functional investigation.

The pronounced elevation of BCAAs—isoleucine and leucine—in the CRC group is consistent with the well-documented reprogramming of amino acid metabolism in malignant cells. BCAAs serve as nitrogen donors for nucleotide biosynthesis and as potent activators of the mechanistic target of rapamycin complex 1 (mTORC1) signaling pathway, which plays a central role in promoting cell proliferation, survival, and metabolic adaptation in cancer [24]. Elevated fecal BCAA levels have been reported across multiple CRC metabolomics studies employing diverse analytical platforms, and their identification in the present dataset further supports the hypothesis that BCAA dysregulation constitutes a robust and reproducible metabolic signature of colorectal malignancy.

Among the individual metabolite biomarkers, furoic acid demonstrated particularly remarkable diagnostic performance, achieving a perfect AUC of 1.000. The lowest CRC value exceeded the highest control value, creating a distinct “biomarker gap” that permits unambiguous classification within this cohort. Furoic acid is a furan derivative primarily generated through the microbial degradation of dietary fibers and pentose sugars. Its exceptional discriminatory capacity in this cohort may reflect disease-associated shifts in the gut microbial community composition and metabolic output. However, a perfect AUC obtained in a relatively small cohort (n = 76) warrants cautious interpretation, as it may reflect overfitting or characteristics unique to this particular cohort. Independent validation in larger, geographically diverse cohorts will be needed before any clinical claims can be supported.

Palmitic acid and tyramine also exhibited outstanding diagnostic performance, with AUC values of 0.996 and 0.980, respectively. Palmitic acid, the most abundant saturated fatty acid in mammalian tissues, has been implicated in CRC pathogenesis through its role in promoting inflammatory signaling via Toll-like receptor 4 (TLR4) activation, lipotoxicity, and epigenetic reprogramming [25]. A recent state-of-the-art review identified palmitic acid as consistently upregulated in stool samples from CRC patients across multiple independent studies [11]. Tyramine, a biogenic amine produced by microbial decarboxylation of tyrosine, has been associated with gut dysbiosis and has been demonstrated to modulate intestinal permeability and inflammatory responses, both of which are implicated in CRC development.

The sole downregulated metabolite, aminoisobutyric acid, warrants particular attention. Its depletion in the CRC group, coupled with its distinctive negative SHAP contribution pattern, suggests a potential protective role in colorectal homeostasis. Beta-aminoisobutyric acid (β-AIBA) has been characterized as a PGC-1α-regulated myokine associated with enhanced mitochondrial function, white fat browning, hepatic β-oxidation, and improved insulin sensitivity [26]—all of which are antagonistic to the metabolic milieu that favors tumorigenesis. The inverse relationship between aminoisobutyric acid concentration and CRC classification probability, as revealed by SHAP analysis, provides a mechanistically coherent interpretation and highlights the capacity of XAI techniques to uncover metabolites with biologically meaningful protective significance.

From a methodological perspective, the superior performance of TPOT (nested CV: AUC = 0.997; MCC: 0.957; hold-out: accuracy = 0.981; AUC = 0.998; MCC = 0.963) relative to LightGBM and XGBoost highlights the practical value of automated pipeline optimization in metabolomics-based classification tasks. The genetic programming approach employed by TPOT enables systematic exploration of a vast combinatorial space of preprocessing steps, feature transformations, and model architectures that would be impractical to evaluate manually. The slightly higher variability observed with LightGBM is consistent with its known sensitivity to hyperparameter settings in small-sample scenarios, whereas XGBoost demonstrated a favorable balance between predictive accuracy and generalization stability [14].

To contextualize the performance of the proposed framework, the classification metrics obtained in the present study were benchmarked against published non-invasive CRC detection modalities. In the literature, the fecal immunochemical test (FIT), which represents the current first-line non-invasive screening tool, has been reported in meta-analytic syntheses to achieve a pooled sensitivity of approximately 76–79% and a specificity of 93–94% for CRC in average-risk populations [27,28]. Microbiome-based machine learning classifiers leveraging fecal shotgun metagenomics have likewise been evaluated across geographically and technically diverse multi-cohort meta-analyses [29,30], with models trained across pooled datasets achieving a mean area under the receiver operating characteristic curve (AUC) of approximately 0.84 in independent leave-one-dataset-out validation [29], and independent external metagenomic validation cohorts yielding AUROC values ranging from 0.69 to 0.91 (interquartile range: 0.79–0.87) [31]. Bulk fecal metabolomic machine learning approaches have yielded AUC values of approximately 0.80 when metabolite panels were employed in isolation, increasing to 0.94 upon integration with microbial signatures [32], while integrative plasma and fecal metabolomic frameworks applied across multiple independent cohorts have demonstrated diagnostic AUC values of 0.848–0.987 [33]. The nested cross-validation AUC of 0.997 achieved by the TPOT pipeline in the present cohort therefore compares favourably with these established benchmarks, suggesting that EV-derived metabolite profiling, when coupled with automated pipeline optimization and dual-layer explainability, may offer incremental discriminatory value over conventional fecal-based screening modalities. It must be emphasized, however, that paired FIT measurements were not available within the present cohort, precluding a direct within-subject comparison; prospective studies incorporating both modalities in the same individuals will be required to formally quantify the incremental diagnostic gain of the proposed framework over FIT and to clarify whether the two assays should be regarded as complementary rather than competing tools. The reproducibility of the present findings is further supported by their concordance with the predominant directional trends reported across independent CRC metabolomic cohorts—including the elevation of branched-chain amino acids, aromatic amino acids, and microbially derived metabolites in CRC-associated gut metabolomes [32]—thereby reinforcing the biological plausibility of the identified panel beyond the boundaries of any single dataset.

The complementary application of SHAP and LIME provided a comprehensive interpretability framework operating at both global and local levels. At the global level, SHAP analysis revealed that furoic acid, palmitic acid, and tyramine are the dominant drivers of CRC classification across the entire cohort, while metabolites such as ethanolamine, lysine, and alanine contribute minimally despite reaching univariate significance. This discrepancy between univariate statistical significance and multivariate predictive importance is a familiar pattern in high-dimensional omics data and highlights the value of ML-based feature importance assessment over univariate screening alone. At the local level, LIME analysis corroborated the SHAP findings by confirming that the same metabolite signatures consistently underlie the model’s classification decisions across individual patients. The concordance between these two independent interpretability methods reinforces confidence that the identified metabolite panel captures biologically and clinically meaningful variation.

From a clinical standpoint, the high discriminatory performance of the identified metabolite panel—particularly furoic acid, palmitic acid, and tyramine—suggests potential utility as a non-invasive, stool-based companion diagnostic that could complement existing screening modalities such as FIT. A metabolomics-based test derived from fecal microbial EVs could be particularly valuable in settings where colonoscopy access is limited, offering a molecularly informed triage step prior to invasive endoscopic evaluation. The transparency afforded by the SHAP and LIME interpretability framework may further facilitate clinical acceptance, as clinicians can inspect which metabolites drove each individual classification decision—a feature increasingly demanded by regulatory bodies for AI-based diagnostic tools [18].

This study has several limitations that merit consideration. First, the relatively modest sample size (n = 76) constrains the statistical power and limits the generalizability of the findings. In particular, biomarker-discovery and metabolite-prediction studies in oncology generally benefit from cohorts of several hundred participants to obtain stable effect-size estimates and to mitigate the well-documented risk of overfitting that arises when high-dimensional metabolomic features are evaluated against a comparatively small number of cases. The metabolite-level discrimination metrics reported here—including the perfect AUC observed for furoic acid—should therefore be interpreted as exploratory point estimates derived from a relatively small cohort, rather than as definitive evidence of clinical utility, and may not be fully reproducible in larger, more heterogeneous populations. Although the 100-repeat hold-out validation strategy was employed to mitigate overfitting and provide robust performance estimates, external validation in independent, geographically and ethnically diverse, and larger cohorts remains an essential next step. Second, the cross-sectional design precludes assessment of temporal changes in metabolite profiles during disease progression or treatment response, and prospective longitudinal studies are needed to determine the prognostic and predictive utility of the identified biomarkers. While the XAI analyses provide valuable insights into the directional contributions of individual metabolites, they do not establish causal relationships, and functional validation through in vitro and in vivo studies is warranted to elucidate the mechanistic roles of the identified metabolites in CRC pathogenesis. The potential confounders including dietary habits, medication use, and microbiome composition were not fully controlled in this analysis, and future studies should incorporate these covariates to enhance the specificity of the identified biomarker signatures. Third, the perfect classification performance of furoic acid (AUC = 1.000) reflects complete separation within this specific cohort and may not generalize to independent populations with different dietary patterns, disease stages, or demographic characteristics. Furthermore, the absence of paired fecal immunochemical test (FIT) measurements in the present cohort precluded a direct within-subject comparison of the EV-metabolomic signature against the current standard non-invasive screening modality; subsequent prospective evaluations should incorporate paired FIT and EV-metabolomic profiling in the same individuals to formally quantify the incremental diagnostic gain of the proposed framework and to determine whether the two assays should be deployed as complementary rather than competing tools. In addition, the present analysis was deliberately restricted to the metabolomic cargo of microbial EVs, and forthcoming work integrating complementary proteomic, transcriptomic, and lipidomic dimensions of the EV cargo is anticipated to further enhance both diagnostic accuracy and mechanistic insight. Finally, it should be acknowledged that the perfect AUC obtained the TPOT pipeline in the present cohort may, in part, reflect cohort-specific characteristics; replication in independent multi-center cohorts will therefore be essential before any clinical claims can be substantiated.

## 4. Materials and Methods

### 4.1. Study Design, Patient Population, and Metabolomics Data

This study utilized a publicly available metabolomic dataset comprising stool-derived samples from a total of 76 participants, including 36 individuals with a confirmed histopathological diagnosis of CRC and 40 healthy volunteers serving as controls. Although the two groups were not formally pair-matched on a one-to-one basis, the CRC patients (mean age ≈ 60 years; range 35–82; ~58% male) and healthy controls (mean age ≈ 58 years; range 30–78; ~55% male) were drawn from a comparable adult population, with no statistically significant difference in age (*p* > 0.05) or sex distribution (*p* > 0.05) between the groups, thereby minimizing the contribution of these demographic factors as confounders of the metabolomic signatures. For the patient group, clinical and demographic variables recorded at the time of enrollment included age, sex, disease stage, tumor localization, and carcinoembryonic antigen (CEA) levels. Healthy control subjects were adults with no history of chronic illness and normal findings on routine laboratory examination. Individuals were excluded from the control group if they carried a prior or current diagnosis of bowel disease, were receiving pharmacological treatment for gastrointestinal conditions, or had a previous history of CRC.

Exclusion criteria applied to both groups included postoperative CRC recurrence, ongoing or recently completed chemotherapy, concurrent diagnosis of another malignancy or metabolic disorder, and use of any systemic medication or antibiotic therapy within one month prior to sample collection. Stool samples were collected from patients prior to surgical intervention or bowel preparation. The day before sample collection, all participants followed a standardized mild diet consisting of low-fat, low-fiber, easily digestible foods. Samples were taken from the midsection of each stool sample using a sterile cotton swab and stored at −20 °C until processing.

Microorganism-derived EVs were isolated from the fecal matrix using a standardized incubation and differential centrifugation-based protocol. Briefly, approximately 100 mg from each fecal sample was homogenized in sterile phosphate-buffered saline and incubated to release the vesicular structures into the aqueous phase. The homogenate was first subjected to low-speed centrifugation (10,000× *g*, 10 min, 4 °C) to precipitate bacterial cells, large food debris, and host cell fragments, and the resulting supernatant was passed through a sterile 0.22 µm membrane filter to remove residual particulate matter and intact microorganisms. The filtered supernatant was then ultracentrifuged at 150,000× *g* for 2 h at 4 °C to precipitate the EV fraction; The resulting pellet was washed and resuspended in sterile PBS to obtain a purified microbial EV preparation used as the input matrix for metabolomic profiling. Following thawing of the frozen EV fractions, metabolomic profiling was carried out using both liquid chromatography coupled to quadrupole time-of-flight mass spectrometry (LC/QTOFMS) and gas chromatography–time-of-flight mass spectrometry (GC/TOFMS), enabling comprehensive coverage of a broad range of polar and non-polar metabolite classes. Metabolite identification confidence was initially assigned at Metabolomics Standards Initiative (MSI) Level 2 for all included metabolites. This study was conducted in full accordance with the ethical principles outlined in the Declaration of Helsinki. Ethical approval was granted by the Inonu University Non-Interventional Clinical Research Institutional Review Board (Decision No: 2026/9776). Based on a preliminary internal analysis yielding a pilot AUC of 0.95 for furoic acid, a two-sided type I error of 0.05, power of 0.80, and a null hypothesis AUC of 0.70, a minimum of 28 CRC patients and 28 controls were required [34]. Our cohort (36 CRC, 40 controls) exceeds this minimum. This study is reported following the TRIPOD [21] and STROBE-metabolomics [35] guidelines.

The rationale for analyzing microbial EVs rather than bulk stool is biological and analytical: fecal EVs selectively encapsulate microbial- and host-derived metabolites in a stable, membrane-protected lumen, which (a) buffers the cargo against the strong matrix effects, oxidation, and enzymatic degradation typical of whole stool, (b) enriches signal originating from microbially active subpopulations directly engaged in host crosstalk, and (c) yields a less variable, more reproducible chromatographic background than crude fecal extracts. In accordance with the Minimal Information for Studies of Extracellular Vesicles (MISEV2018) guidelines, EV characterization in the source dataset was confirmed through complementary analytical procedures, including transmission electron microscopy for morphological verification and nanoparticle-tracking analysis for size distribution and particle-concentration quantification. EV preparations exhibited the expected size range (30–200 nm) and morphological features characteristic of bacterial outer-membrane vesicles, while protein co-isolation was monitored via bicinchoninic acid (BCA) assay to ensure consistent particle-to-protein ratios across samples. Although microbial EVs harbour a heterogeneous molecular cargo comprising proteins, nucleic acids (mRNA, miRNA), lipids, and small-molecule metabolites, the present investigation deliberately focused on the metabolomic compartment for three principal reasons: (i) metabolites represent the most downstream readout of microbial functional activity and therefore reflect the most proximal disease-relevant biochemical phenotype; (ii) untargeted LC/QTOFMS and GC/TOFMS profiling provides comprehensive, quantitative, and reproducible coverage of polar and non-polar low-molecular-weight species within a single analytical workflow; and (iii) prior literature indicates that EV-encapsulated metabolite signatures yield superior between-group discrimination relative to bulk fecal metabolomic profiling, while remaining compatible with rapid, scalable, and cost-effective clinical assays. Future multi-omics integration of EV-derived proteomic and transcriptomic cargo with the present metabolomic signature is nonetheless anticipated to further refine diagnostic accuracy and mechanistic interpretability.

### 4.2. Data Preprocessing and Statistical Analysis

Prior to scaling, raw peak intensities were processed through a sequential pre-analytical pipeline to ensure data quality and inter-sample comparability. Missing values were imputed using a k-nearest neighbours algorithm (k = 5) operating on feature-level similarity, which preserves the underlying distributional properties more faithfully than half-minimum substitution. Log2 transformation was applied to stabilize variance. Given that all samples were acquired within a single analytical sequence with QC monitoring (CV < 30%), sample-level normalization was deemed unnecessary. Because all samples were acquired within a single analytical sequence in the source dataset, formal cross-batch correction algorithms (e.g., ComBat or QC-RLSC) were not required.

For univariate statistical analysis, preprocessing was applied to the full dataset, as these transformations are unsupervised and do not access outcome labels. For all machine learning analyses, preprocessing was applied within each fold as described in Section 4.3. Univariate comparisons of individual metabolite concentrations between the CRC and control groups were performed using the Mann–Whitney U test, given the non-normal distribution of most metabolomic features as confirmed by the Shapiro–Wilk test. Fold change (FC) was calculated as the ratio of median metabolite concentrations in the CRC group relative to controls, and log2 fold change (Log2FC) values were derived accordingly. The statistical significance threshold was set at *p* < 0.05 after false discovery rate (FDR) correction using the Benjamini–Hochberg procedure to account for multiple comparisons. Metabolites with FDR-corrected *p*-values < 0.05 were considered significantly dysregulated, regardless of fold change magnitude.

The diagnostic performance of individual metabolites was evaluated through ROC analysis, with the AUC serving as the primary metric of discriminatory capacity. For each significant metabolite, optimal sensitivity and specificity were determined using the Youden index, and 95% confidence intervals (CIs) for AUC values were estimated via the DeLong method.

### 4.3. Machine Learning-Based Classification

To develop an optimized predictive model for CRC classification, three supervised ML algorithms were implemented and compared: TPOT, LightGBM, and XGBoost. These approaches were selected for their proven ability to handle high-dimensional biological datasets of limited sample size and their built-in regularization mechanisms.

TPOT (version 1.1) was employed as an AutoML framework that leverages genetic programming to optimize the entire ML pipeline, using roc_auc as the optimization objective and a maximum search time of 5 min per repeat. LightGBM was configured with gradient-based one-side sampling (GOSS) and exclusive feature bundling (EFB). XGBoost was trained with L1 and L2 regularization to mitigate overfitting. Hyperparameter optimization for LightGBM and XGBoost was performed via Bayesian optimization using Optuna (50 trials per model per repeat) with an inner 5-fold cross-validation on the training partition. The hyperparameter search spaces were as follows: LightGBM—num_leaves [7,15,31], learning_rate [0.01, 0.05, 0.1], n_estimators [100–500], max_depth [2–5], min_child_samples [20–60], subsample [0.4–0.7], colsample_bytree [0.3–0.6], reg_alpha [0.1–20.0] (log-scale), reg_lambda [0.1–20.0] (log-scale), min_split_gain [0.0–1.0]; XGBoost—max_depth [2–8], learning_rate [0.01–0.3] (log-scale), n_estimators [100–600], subsample [0.5–1.0], colsample_bytree [0.5–1.0], reg_alpha [0.001–2.0] (log-scale), reg_lambda [0.001–2.0] (log-scale), min_child_weight [1–5], gamma [0.0–1.0].

Model performance was evaluated using a 100-repeat stratified hold-out validation approach, whereby the dataset was randomly partitioned into training (80%) and testing (20%) subsets while maintaining the original class distribution in each repetition. In addition, nested stratified 5-fold cross-validation repeated 100 times was implemented to provide unbiased performance estimates. The outer 5-fold loop was used for performance estimation and the inner optimization (Optuna or TPOT search) operated exclusively on the training partition of each outer fold.

Preprocessing steps (KNN imputation with k = 5, log2 transformation, and Pareto scaling) were applied within each cross-validation fold and each hold-out repeat. The imputer and scaling parameters were fitted exclusively on the training partition and subsequently applied to the test partition using a held-out transform step, ensuring complete separation between training and test data at every stage of the pipeline and eliminating data leakage. Performance metrics computed for each model included accuracy, precision, sensitivity, F1-score, AUC, Brier score, and Matthews Correlation Coefficient (MCC). The Brier score was included as a measure of probabilistic calibration, while the MCC was prioritized as a balanced evaluation metric given its demonstrated superiority over accuracy and F1-score in binary classification with imbalanced data [36]. All ML analyses were implemented in Python (version 3.11) using scikit-learn (v1.8.0), LightGBM (v4.6.0), XGBoost (v3.2.0), TPOT (v1.1.0), and Optuna (v4.7.0) libraries. The complete pipeline is available at https://github.com/drhilal/CRC-study, (accessed on 2 June 2026).

### 4.4. Explainable Artificial Intelligence: SHAP and LIME

To enhance the interpretability of the best-performing ML model, two complementary post hoc explainability techniques were applied. SHAP analysis was performed to quantify the global contribution and directional effect of each metabolite on model predictions. SHAP values, grounded in cooperative game theory, decompose each individual prediction into additive feature contributions, thereby enabling both cohort-level (global) and sample-level (local) interpretation of the model’s decision-making process [19]. The resulting beeswarm plots were used to visualize the distribution of SHAP values across all samples, with color encoding indicating observed feature values (high versus low concentration).

LIME analysis was subsequently applied to characterize feature contributions at the level of individual predictions. LIME approximates the behavior of a complex classification model locally around each sample by constructing an interpretable surrogate model—typically a sparse linear regression—within a perturbed neighborhood of the instance of interest [20]. This approach provides patient-level insight into which metabolites most strongly drove each specific classification decision, thereby complementing the global perspective afforded by SHAP. The combined application of SHAP and LIME was intended to deliver a dual-layer interpretability framework that bridges aggregate model behavior with individual-level clinical relevance. SHAP analysis was performed on the held-out test partition of the final stratified split. The TPOT pipeline was trained on the corresponding training partition and applied to the test set for SHAP value computation, ensuring no information from the test set influenced the model or preprocessing parameters.

## 5. Conclusions

This study demonstrates that the metabolomic profiling of stool-derived microbial EVs, when integrated with optimized ensemble ML classifiers and dual-layer XAI, provides a highly accurate, transparent, and clinically informative framework for non-invasive CRC detection. Within the present cohort, the identified panel of fecal microbial EV-derived metabolites—most prominently furoic acid, palmitic acid, and tyramine—exhibited strong predictive capacity for distinguishing CRC patients from healthy controls and therefore represents a biologically plausible, non-invasive candidate biomarker signature for CRC. We propose that this metabolite panel can serve as a hypothesis-generating predictive marker set for CRC; however, given the limited sample size and single-cohort design, this predictive capacity must be regarded as preliminary and should not be interpreted as evidence of standalone clinical utility prior to large-scale, prospective, multi-center validation. The identified metabolite panel—anchored by furoic acid, palmitic acid, and tyramine as the most discriminative and predictive biomarkers—achieved near-perfect classification performance across three independent algorithms, with TPOT emerging as the optimal pipeline. Nested cross-validation demonstrated robust performance: TPOT achieved AUC = 0.997 ± 0.005, sensitivity = 0.973 ± 0.022, and MCC = 0.957 ± 0.033. Hold-out validation corroborated these findings (AUC = 0.998 ± 0.008). SHAP and LIME analyses provided biologically coherent and clinically interpretable explanations of model behavior, while the distinctive protective pattern of aminoisobutyric acid illustrates the capacity of XAI techniques to reveal metabolites with meaningful biological significance beyond conventional statistical screening. Future research should prioritize external validation in large, prospective, multi-center cohorts, and functional mechanistic studies, to advance these findings toward clinical implementation as a non-invasive CRC screening tool.

## Figures and Tables

**Table 1:** Differentially expressed metabolites between CRC and control groups with corresponding fold change (FC), log2 fold change (Log2FC), statistical significance (−log10[*p*]), Benjamini–Hochberg adjusted *p*-value, regulation direction, area under the ROC curve (AUC), 95% confidence interval (CI), sensitivity, and specificity.

Metabolite	FC	Log2FC	−log10*p*	Adj_*p*	Regulation	AUC	CI_95 (AUC)	Sensitivity	Specificity
Furoic acid	1.433	0.519	13.146	<0.001	Up	1.000	1.000–1.000	1.000	1.000
Palmitic Acid	1.447	0.533	12.940	<0.001	Up	0.996	0.986–1.000	1.000	0.925
Tyramine	1.592	0.671	12.200	<0.001	Up	0.981	0.948–1.000	0.944	0.950
Phenol	1.476	0.562	11.515	<0.001	Up	0.966	0.928–0.994	0.917	0.925
Oxalic acid	4.038	2.013	8.587	<0.001	Up	0.898	0.825–0.961	0.750	0.950
Succinic acid	4.523	2.177	6.619	<0.001	Up	0.845	0.742–0.932	0.889	0.750
Leucine	2.059	1.042	6.030	<0.001	Up	0.828	0.726–0.918	0.750	0.825
Isoleucine	2.149	1.104	5.690	<0.001	Up	0.817	0.717–0.907	0.778	0.775
Hexanoic acid	1.385	0.470	5.166	<0.001	Up	0.801	0.690–0.891	0.611	0.825
Oleic Acid	1.320	0.401	4.510	<0.001	Up	0.778	0.668–0.877	0.778	0.750
Alanine	1.805	0.852	3.470	<0.001	Up	0.740	0.622–0.846	0.889	0.500
Ethanolamine	1.572	0.652	2.424	0.005	Up	0.694	0.568–0.812	0.556	0.800
Aminoisobutyric acid	0.947	−0.079	2.310	0.006	Down	0.312	0.198–0.442	0.972	0.050
Lysine	1.419	0.505	1.870	0.014	Up	0.665	0.533–0.781	0.500	0.825

**Table 2:** Comparative classification performance of TPOT, LightGBM, and XGBoost models evaluated via 100-repeat stratified hold-out validation. Values are reported as mean ± standard deviation. AUC, area under the receiver operating characteristic curve; MCC, Matthews Correlation Coefficient.

Model	AUC	Accuracy	Precision	Sensitivity	F1	Brier	MCC
TPOT	0.998 ± 0.008	0.981 ± 0.036	0.986 ± 0.040	0.976 ± 0.055	0.980 ± 0.037	0.026 ± 0.038	0.963 ± 0.069
LightGBM	0.855 ± 0.228	0.846 ± 0.223	0.702 ± 0.452	0.700 ± 0.452	0.700 ± 0.451	0.109 ± 0.101	0.693 ± 0.447
XGBoost	0.997 ± 0.012	0.976 ± 0.040	0.990 ± 0.033	0.964 ± 0.069	0.975 ± 0.042	0.034 ± 0.029	0.955 ± 0.074

**Table 3:** Nested cross-validation performance (mean ± SD over 100 repeats × 5 folds). AUC, area under the receiver operating characteristic curve; MCC, Matthews Correlation Coefficient.

Model	AUC	Sensitivity	MCC
TPOT	0.997 ± 0.005	0.973 ± 0.022	0.957 ± 0.033
LightGBM	0.876 ± 0.098	0.740 ± 0.197	0.727 ± 0.194
XGBoost	0.995 ± 0.006	0.968 ± 0.015	0.964 ± 0.016

## Data Availability

The publicly available metabolomics data, complete analysis pipeline, per-repeat validation results, and TPOT pipeline selection frequencies supporting the findings of this study are available at https://github.com/drhilal/CRC-study (files: df.xlsx, crc_final_last.py, holdout_checkpoint.json, TPOT_pipeline_frequencies.csv, accessed on 2 June 2026).
